# The Multifaceted Opportunities Provided by the Pheomelanin-Inspired 1,4-Benzothiazine Chromophore: A Still-Undervalued Issue

**DOI:** 10.3390/molecules28176237

**Published:** 2023-08-25

**Authors:** Maria Laura Alfieri, Lucia Panzella

**Affiliations:** Department of Chemical Sciences, University of Naples “Federico II”, I-80126 Naples, Italy; marialaura.alfieri@unina.it

**Keywords:** 1,4-benzothiazine, pheomelanin, 2-aminothiophenol, acidichromism, electrochromism, pH sensing, smart packaging, bioimaging, photocatalysis

## Abstract

1,4-Benzothiazines are the main building blocks of the naturally occurring pheomelanin pigments, and their chromophoric properties have been strongly related to the well-known phototoxicity of these pigments, partly responsible for the high incidence of melanoma and other skin cancers in red-haired people. However, some peculiar features of the 1,4-benzothiazine chromophore could be functionally exploited in several sectors. Within this context, in this perspective, an overview of the very recently reported applications of the 1,4-benzothiazine chromophore in pH sensing, filter permeability control, smart packaging, electrochromic device fabrication, bioimaging, photocatalysis, and HPLC detection systems is provided, together with a brief presentation of recently developed synthetic approaches to the 1,4-benzothiazine scaffold, with the aim of emphasizing the still-undervalued multifunctional opportunities offered by this class of compounds.

## 1. Introduction

1,4-Benzothiazines have been the focus of several research papers and review articles, mainly aimed at the synthesis of new compounds for potential or overt applications, primarily in the medicinal sector, due their potent and countless pharmacological and biological properties. These include mainly antihypertensive, antipsychotic, antidiabetic, anticancer, anti-inflammatory, antimalarial, antiviral, antimicrobial, antitubercular, and analgesic activities. Most of these properties, which are generally associated with the ability to inhibit specific enzymes, have been found to be strictly related to the unique nitrogen and sulfur relative arrangement in the benzothiazine scaffold [1,2,3,4,5,6]. Some papers have also reported the use of 1,4-benzothiazines as dyestuffs, color photographic developers, corrosion inhibitors, and antioxidants for rubbers and elastomers [1].

**Figure 1:**
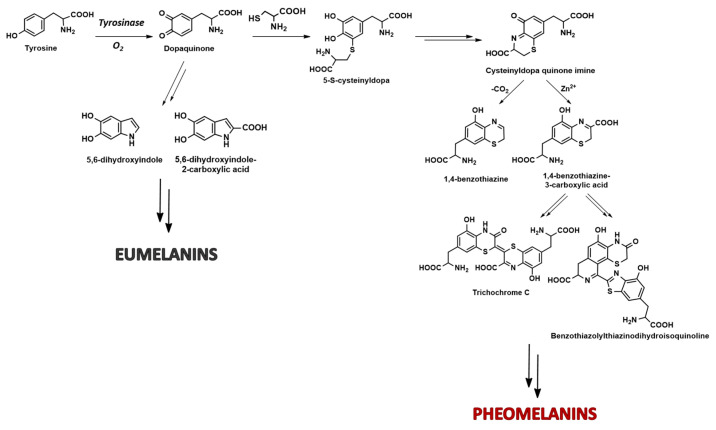
Schematic overview of eumelanin and pheomelanin biosynthesis.

As for the natural occurrence, 1,4-benzothiazines represent the main structural units of an important class of natural pigments, that is, pheomelanins [7,8,9,10,11,12,13,14], as recently demonstrated through the use of biomimetic synthetic models [15,16]. These latter ones are the yellow/reddish pigments typical of red-haired individuals of Celtic origin, biosintetically produced as a consequence of a genetically induced switch of the “normal” melanocyte activity, leading to dark photoprotective melanin pigments. At the biochemical level, this switch is the result of a lower tyrosinase activity, favoring the concomitant intervention of cysteine in the melanogenesis pathway. Non-enzymatic addition of the SH group to the oxidation product of tyrosine, dopaquinone, leads to the formation of isomeric cysteinyldopas [9,17,18,19,20]. As a result, the intramolecular cyclization pathway of dopaquinone to 5,6-dihydroxyindoles leading to eumelanins is inhibited, and an alternate 1,4-benzothiazine route to pheomelanins as well as trichochromes becomes dominant (Figure 1). Indeed, the oxidation of cysteinyldopas followed by intramolecular cyclization leads to a quinone imine, which can either undergo decarboxylation or isomerize without decarboxylation to give a 1,4-benzothiazine or its 3-carboxylic acid derivative, respectively (Figure 1). This last pathway is particularly favored in the presence of Zn^2+^ ions, a trace element abundant in skin and hair [21]. The benzothiazine units, particularly the carboxylated ones and the related compounds trichochrome C and the benzothiazolylthiazinodihydroisoquinoline shown in Figure 1, are the main ones responsible of the well-known phototoxicity of pheomelanins, conferring the pigments pronounced UVA and visible absorption features, accounting for light-dependent reactive oxygen species production [21,22,23]. Indeed, pheomelanosomes exhibit a photoionization threshold falling in the UVA region of the solar spectrum (at ca. 326 nm) [23] and, further, to excitation to the singlet state, the pheomelanin chromophore decays to the triplet state with transfer of an electron to oxygen, leading to the generation of a superoxide anion [24].

Starting from these observations, it is clear that the 1,4-benzothiazine structural motif exhibits very peculiar chromophoric properties. These latter properties, including also photochromism and acidichromism [9,25,26,27], coupled with the chemical versatility of the benzothiazine ring allowing for further functionalization, could actually be easily exploited for the implementation of smart devices, including sensors, photocatalysts, and bioimaging systems [28]. However, the multifaceted opportunities provided by the 1,4-benzothiazine chromophore remain still, in part, underexploited.

Within this context, this perspective will provide an overview of the potential applications of the 1,4-benzothiazine chromophore, as appeared in the literature in the last three years. Recently developed synthetic strategies of 1,4-benzothiazine systems and related compounds will also be briefly presented, with a particular focus on green approaches.

## 2. Recent Synthetic Approaches to 1,4-Benzothiazines

1,4-Benzothiazines are heterocyclic compounds consisting of a benzene ring attached to the 6-membered heterocycle 1,4-thiazine. The name is applied to both the 2*H*- and 4*H*-isomers of the molecule. Several synthetic strategies have been reported in recent years that provide easy access to variously functionalized 1,4-benzothiazines, also from a green chemistry perspective [29]. These strategies are based on different chemical approaches, mainly involving the coupling of 2-aminothiophenols with carbonyl compounds. Other protocols have also been described involving, e.g., coupling of thiophenols with α-halohydroxamates, oxidative ring expansion of benzothiazoles, and oxidative C–H functionalization with elemental sulfur.

### 2.1. Coupling of 2-Aminothiophenols with Carbonyl Compounds

2-Aminothiophenol undoubtedly represents one of the main synthetic precursors of 1,4-benzothiazines, as recently reviewed in several papers, and α-bromo carbonyl compounds, such as bromopyruvates or phenacyl bromides, as well as dicarbonyl compounds, are commonly employed as the electrophilic counterpart [30,31,32]. Current research is, therefore, particularly devoted to the development of green and biocompatible reaction conditions for the implementation of synthetic strategies based on the use of this compound, as demonstrated by the numerous papers that have fleshed out, in the last three years, the very poor relevant scenario (only three examples) dating back seven years [4].

**Figure 2:**
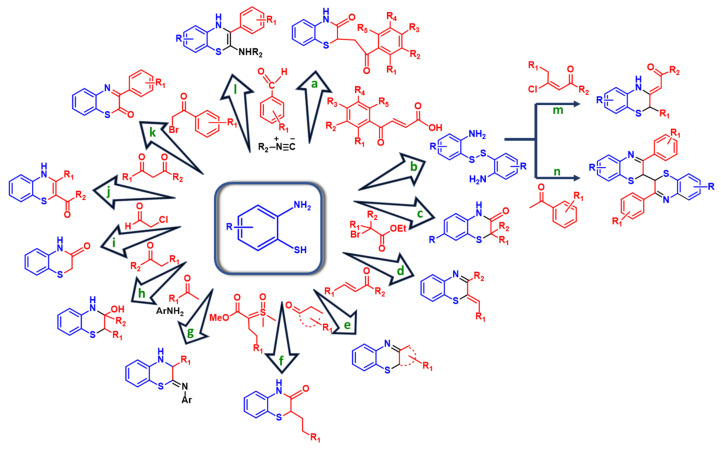
Overview of the synthetic approaches to 1,4-benzothiazines by coupling of 2-aminothiophenols with carbonyl compounds.

As an example, the possibility to run the reactions of substituted 2-aminothiophenols with 1,3-dicarbonyl compounds under metal-free conditions using polyethyleneglycol (PEG-200) as a solvent has been demonstrated (Figure 2, path j). Isolation yields > 75% were reported for reactions run at 80 °C using equimolar amounts of the reactants [33].

On the same line, eco-friendly approaches based on the reaction of 2-aminothiophenol with an aldehyde and isocyanide in ethanol/water under visible light irradiation have been recently reviewed (Figure 2, path l). In particular, yields > 90% were observed even in the absence of photocatalysists under 22 W irradiation with compact fluorescence light for 4.5 h [34].

The same applies to mechanochemical protocols involving the reaction of 2-aminothiophenols with 2-bromoalkanoates, although, in this case, benzothiazinones were obtained (Figure 2, path c). KF/Al_2_O_3_ was used as a catalyst, leading to differently substituted benzothiaziones in yields > 75%. In addition, the catalyst was easily recovered at the end of the reaction and efficiently reactivated via microwave heating with no loss of activity for four cycles [35].

1,4-Benzothiazin-3-one has also been prepared by the reaction of 2-aminothiophenol with 2-chloroacetaldehyde. The reaction was run in the presence of TiCl_4_ as Lewis acid and of potassium carbonate as the catalyst in refluxed DMF for 40 min, allowing researchers to obtain the compound in a 99% yield. The benzothiazione was then reacted with aryl/alkyl halide in the presence of potassium carbonate and tetraethylammonium bromide to obtain derivatives with potential anticonvulsant activity (Figure 2, path i) [36].

Benzothiazinones have also been obtained through the reaction of equimolar amounts of 2-aminothiophenol and β-aroylacrylic acids (Figure 2, path a) in the presence of glacial acetic acid under reflux in ethanol for 2–4 h. The products were obtained in pure form in >95% yields via simple crystallization of the precipitate [37].

Solvent-free conditions have also been optimized for the reaction of 2-aminothiophenol with 1,3-dicarbonyl compounds involving the use of graphene oxide as a recyclable and highly efficient (yield > 75%) heterogeneous catalyst (Figure 2, path j) [38], whereas ultrasonication (40 kHz, in THF, at 60 °C, for 2 h) has been proposed for the development of catalyst-free conditions for the reaction of 2-aminothiophenol with phenacyl bromides (Figure 2, path k) [39]. 

A continuous flow system to 1,4-benzothiazines using β-chlorovinyl ketones and 2,2′-dithiodianilines, that is, the oxidized forms of the corresponding 2-aminothiophenols (Figure 2, path b), has also been developed (Figure 2, path m). The optimized reaction conditions were flow rates of 0.02 mL/min for both reagents dissolved in 1,1,2-trichloroethane, a temperature of 80 °C, and a residence time of 150 min. Triethylamine was also needed to activate the β-chlorovinyl ketone. Most of the products were obtained in >55% yield after column chromatography [40]. The microwave-assisted reaction (150 W/125 °C, 15 min) of 2,2′-dithiodianilines with acetophenones using I_2_ in acetonitrile to prepare bibenzo-1,4-benzothiazines has been described too (Figure 2, path n). Also, in this case, isolation yields > 50% were obtained after flash chromatography [41].

The possibility to exploit α,β-unsaturated, cyclic, linear, and fluoroalkyl ketones to synthesize various benzothiazines in good yields has recently been reviewed (Figure 2, paths d, e, h) [42].

Imino 1,4-benzothiazines have also been prepared via the reaction of 2-aminothiophenols with methyl ketones and anilines in chlorobenzene, at 120 °C, for 16 h, in the presence of potassium iodide and oxygen (Figure 2, path g) [43].

The possibility to exploit 2-aminothiophenol for the synthesis of fused benzothiazines, such as pyrido-1,4-benzhothiazines, spirobenzothiazine-pyrroles, and benzothiazinoisoindolones and benzoindenothiazinones, has also been recently reported [44,45,46,47].

Very recently, the S-H insertion reaction of 2-aminothiophenol with α-alkylated sulfoxonium ylides has been employed for the synthesis of 1,4-benzothiazin-3-one derivatives in a 42–78% yield (Figure 2, path f). The reaction was run in acetonitrile at 60 °C for 48 h, leading to the products in 42–78% yields [48].

### 2.2. Other Synthetic Routes

**Figure 3:**
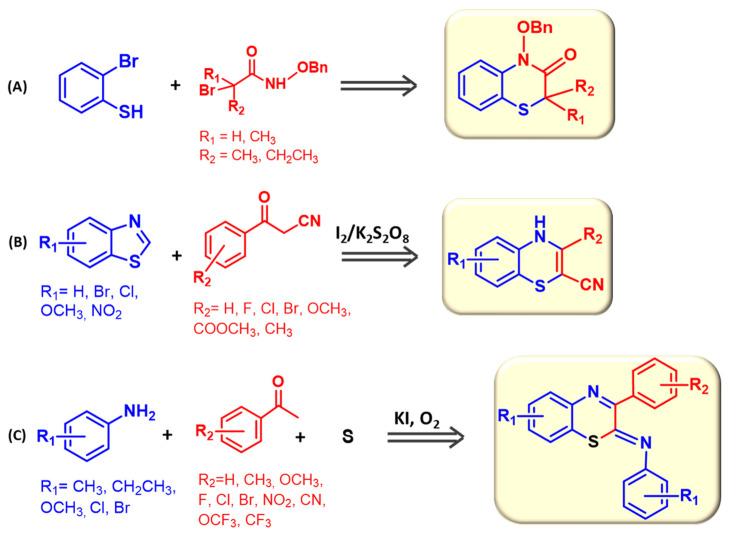
Synthetic approaches to 1,4-benzothiazines based on the use of (**A**) 2-bromothiophenol and α-halohydroxamates, (**B**) benzothiazoles and 3-oxo-3-arylpropanenitriles, or (**C**) acetophenones, anilines, and elemental sulfur (**C**) [49,50,51].

Very recently, an alternative synthetic pathway to the closure of a 1,4-benzothiazine ring has been proposed, involving the reaction of 2-bromothiophenol with α-halohydroxamates (Figure 3A) in 9:1 *v*/*v o*-dichlorobenzene/*tert*-amyl alcohol, at 140 °C, for 7 h. The reaction initially leads to a α-thioamide, which may then undergo cyclization under Pd/Cu catalysis, leading to the desired compounds in a 48–55% yield after purification via column chromatography [49].

Variously substituted 1,4-benzothiazines have also been prepared by I_2_/K_2_S_2_O_8_-promoted ring expansion of benzothiazoles in the presence of 3-oxo-3-arylpropanenitriles (Figure 3B). The reaction was run in methanol at 80 °C for 16 h and led to desired compounds in up to 98% yield [50].

Finally, the reaction of acetophenones, anilines, and elemental sulfur in the presence of KI, DMSO, and oxygen also afforded 1,4-benzothiazines (Figure 3C). The reaction was run using chlorobenzene as the solvent at 150 °C for 16 h [51].

## 3. Exploitation of the Chromophoric Properties of the 1,4-Benzothiazine System

Despite the keen interest in developing new or improving already reported access routes to 1,4-benzothiazine scaffolds, the possibility to exploit their peculiar chromophoric properties has been, to date, significantly underexploited. As reviewed in the following paragraphs, proposed applications of the 1,4-benzothiazine system chromophoric features that have appeared in the literature in the last three years include pH sensing, filter permeability control, smart packaging, electrochromic device fabrication, bioimaging, photocatalysis, and derivatization for HPLC analysis. These generally involve conjugation of the 1,4-benzothiazine moiety with other chromophoric units, leading to highly π-electron-delocalized and, hence, colored compounds, exhibiting chromophoric and, consequently, in some cases, fluorometric features that are at the basis of their possible exploitation in smart systems.

### 3.1. pH Sensing

**Figure 4:**
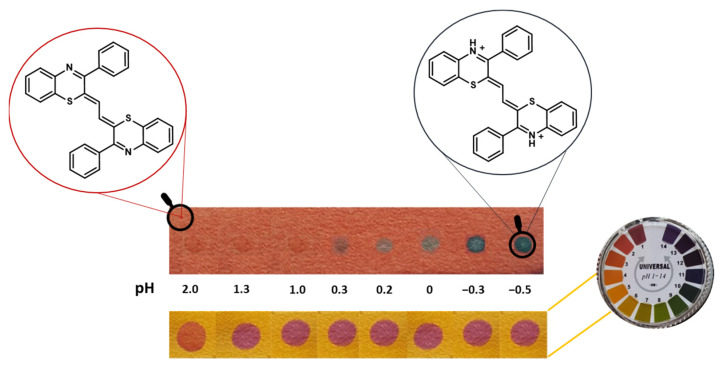
Chromatographic paper dipped in a solution of 2Z,2′Z-(1,2-ethanediylidene)bis(3-phenyl-2*H*-1,4-benzothiazine) as a colorimetric sensor for acidic pHs compared to a universal pH indicator paper [52].

The acidichromic behavior of the 1,4-benzothiazine scaffold chromophore has prompted its possible use for the implementation of visual pH sensors. As a remarkable example, 3-phenyl-2*H*-1,4-benzothiazine, obtained through the reaction of 2-aminothiophenol and phenacyl bromide in anhydrous diethyl ether, was doubly condensed with glyoxal in HCl/acetonitrile at 70 °C by exploiting the high nucleophilicity of the enamine type C-2 position to provide a 2*Z*,2′*Z*-(1,2-ethanediylidene)bis(3-phenyl-2*H*-1,4-benzothiazine) (Figure 4), exhibiting an absorption maximum at ca. 480 nm (red color) at pH ≥ 4.0 and at ca. 610 nm (light blue color) and −640 nm (deep blue color) at lower pH values (pH 2 and pH −0.4, respectively) [52]. A pH sensor paper was, therefore, prepared by dipping chromatographic paper into a solution of the compound, allowing for visual detection of very acidic pHs compared to commercial pH indicators (Figure 4) [52].

**Figure 5:**
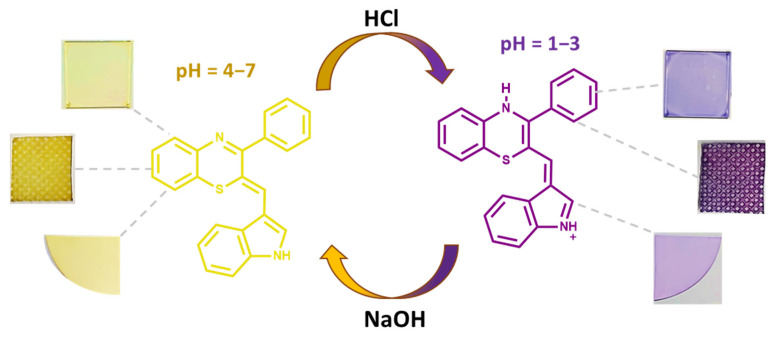
pH sensing via reversible chromophoric changes for the 2-((1*H*-indol-3-yl)methylene)-3-phenyl-2*H*-1,4-benzothiazine) system [53].

Similarly, the condensation products of the same 3-phenyl-2*H*-1,4-benzothiazine with indole-3-carboxaldehyde showed a marked and reversible (up to 15 cycles) acidichromic behavior, with a shift from a yellow (pH > 4) to a purple (pH < 3) color, even when applied as a coating or adsorbed on different materials (glass, nylon, cotton, paper) (Figure 5) [53].

### 3.2. Filter Permeability Control

**Figure 6:**
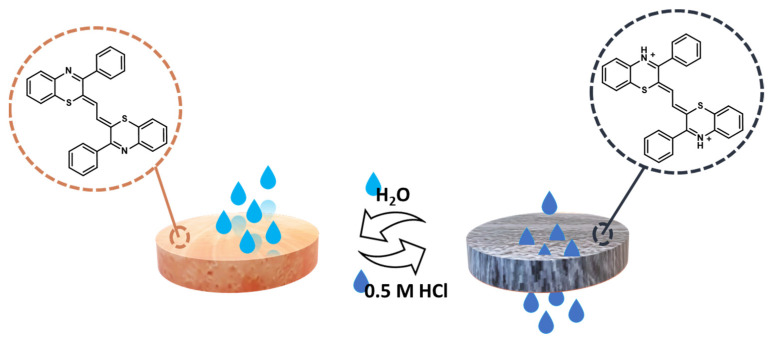
Cellulose acetate filter permeability control through coating with 2*Z*,2′*Z*-(1,2-ethanediylidene)bis(3-phenyl-2*H*-1,4-benzothiazine) [52].

As shown above, the acidichromic behavior of 1,4-benzothiazines is associated with the protonation of the nitrogen atom of the heterocyclic system. This, of course, may also result in a switch of the compound from hydrophobic to hydrophilic, which can be exploited for tuning and monitoring the permeability of filtering material functionalized with a proper 1,4-benzothiazine scaffold. As a proof of concept, a paper filter dipped into a methanolic solution of the (1,2-ethanediylidene)bis(3-phenyl-2*H*-1,4-benzothiazine) described above and left to dry (red in color) was found to be impermeable in water but not acid solutions, which rapidly passes through the filter with the associated color change to blue, allowing one, therefore, to monitor the hydrophobic/hydrophilic state of the device (Figure 6) over several cycles given the high reversibility and robustness of the system compared to other cyanines [52].

### 3.3. Smart Packaging

**Figure 7:**
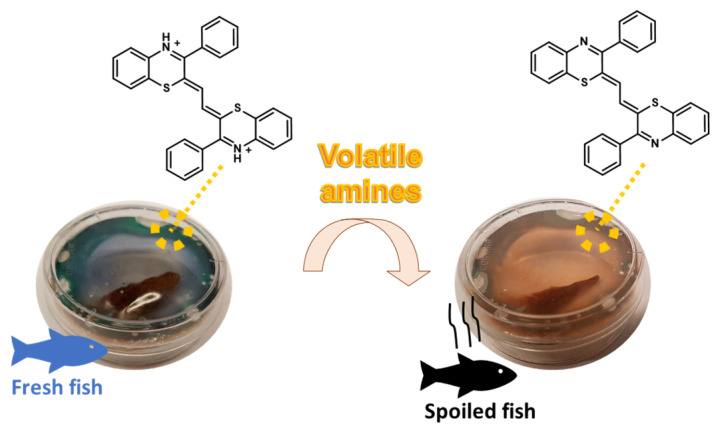
Smart poly(lactic acid) films loaded with the protonated form of 2*Z*,2′*Z*-(1,2-ethanediylidene)bis(3-phenyl-2*H*-1,4-benzothiazine) for detection of decomposing fish fillets [52].

One of the most obvious applications of the acidichromic behavior of 1,4-benzothiazines is, of course, the implementation of food deterioration sensors to be used in smart packaging. As an example, the condensation product of 3-phenyl-2*H*-1,4-benzothiazine with glyoxal described above was also exploited to functionalize poly(lactic acid) films, alginate hydrogels, or chromatographic papers to visually detect volatile amines produced during fish spoilage or organic acids deriving from bacterial spoilage of milk (Figure 7). Although the safety for use in foods still has to be assessed, the high stability and easy availability through a scalable and easy-to-run synthetic procedure makes this compound a promising alternative to natural cyanins for food freshness monitoring [52].

### 3.4. Electrochromic Device Fabrication

**Figure 8:**
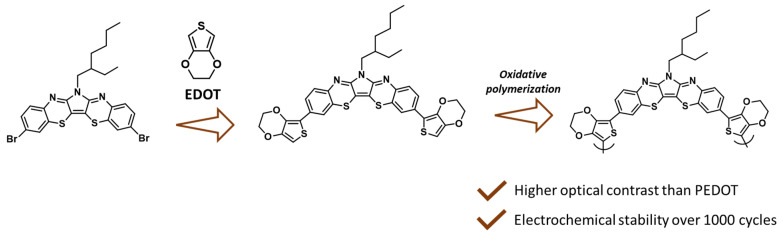
(Pyrrolo)bis(1,4-benzothiazine) TRPZ as a suitable scaffold for electrochromic device fabrication [54].

The possibility to exploit the 1,4-benzothiazine ring to fabricate an electrochromic material has very recently been demonstrated. In particular, a (pyrrolo)bis(1,4-benzothiazine) has been incorporated in a 3,4-ethylenedioxythiophene (EDOT)-based film to facilitate the higher mobility of holes, resulting in an electrochromic material characterized by very fast switching times (4.07 s for coloration and 0.47 s for bleaching at 539 nm) compared to reference polymers, like polyaniline or poly(3,4-ethylenedioxythiophene) (PEDOT). True electrochromic devices were also fabricated, exhibiting an outstanding electrochromic performance, switching from a neutral color state to an oxidized colorless state with a robust cyclic stability over 1000 cycles (Figure 8) [54].

### 3.5. Bioimaging

**Figure 9:**
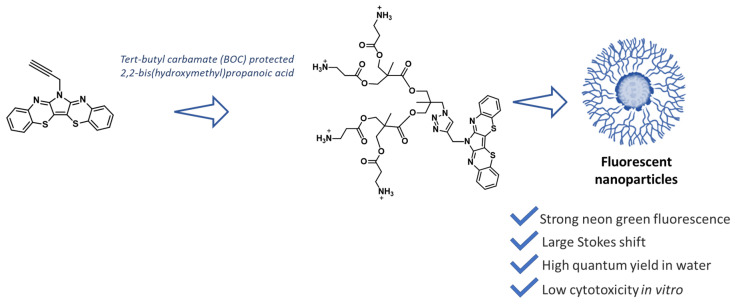
(Pyrrolo)bis(1,4-benzothiazine) TRPZ as a suitable scaffold for fluorescent nanoparticle preparation for applications in diagnostics [55].

A similar (pyrrolo)bis(1,4-benzothiazine) has been used as a scaffold to prepare fluorescent nanoparticles for applications in fluorescence imaging for diagnostics. Indeed, this highly conjugated compound is able to form π-aggregates at high concentrations with a bright-green-yellow fluorescence emission. In particular, the prepared nanoparticles exhibited a strong neon green fluorescence with an emission maximum at 695 nm, with a very large Stoke shift (201 nm) and a high quantum yield (49%) in water, which are both fundamental characteristics for bioimaging applications. They also exhibited promising cellular uptake properties, with very low cytotoxicity (Figure 9) [55].

**Figure 10:**
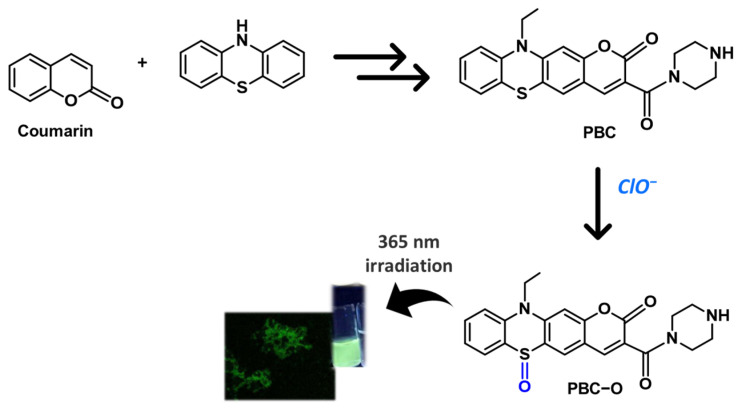
Benzothiazine-fused coumarin derivative for hypochlorite sensing. Photos: confocal fluorescence image of cellular ClO^−^ detected by use of PBC and digital picture of fluorescence response of PBC upon addition of ClO^−^ under 365 nm irradiation [56].

A 1,4-benzothiazine ring fused with a coumarin moiety (PBC) has been, instead, exploited as a fluorescent probe for the bioimaging of cellular hypochlorite. The compound exhibited a weak fluorescence in water, which was enhanced selectively in the presence of hypochlorite as a result of sulfur oxidation (PBC-O) (Figure 10). The high selectivity and sensitivity for ClO^−^ ions compared to other ClO^−^-specific fluorescent probes make this compound very promising for monitoring exogenous and endogenous hypochlorite in living cells [56].

The peculiar spectral features of 1,4-benzothiazines have also been identified as the main features responsible for the possibility to directly visualize neuromelanin in brain tissue via soft X-ray spectromicroscopy, with the aim of providing new advances in the understanding of Parkinson’s disease etiology [57].

### 3.6. Photocatalysis

**Figure 11:**
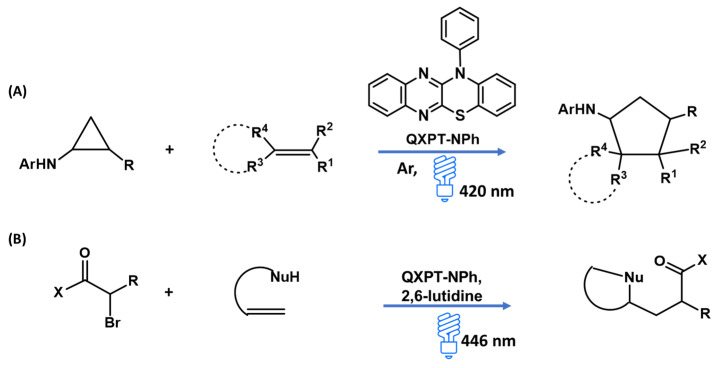
*N*-phenyl quinoxaline-phenothiazine (QXPT-NPh) as a photoredox catalyst in (**A**) the light-mediated [3 + 2] cycloadditions of cyclopropylamines with olefins or (**B**) for the difunctionalization of unactivated olefins through intermolecular addition of α-bromoketones, -esters, or -nitriles [58,59].

A 1,4-benzothiazine moiety fused with a quinoxaline ring (QXPT-NPh) was recently synthesized and evaluated as a photoredox catalyst for the visible-light-mediated [3 + 2] cycloaddition of arylcyclopropylamine and olefins. Even at a concentration as low as 0.1 mol%, reaction yields higher than 75% were observed. The catalytic mechanism would involve the formation of a complex between the arylcyclopropylamine and the catalyst through the establishment of a hydrogen bond. Under visible-light irradiation, this complex would be promoted to the excited state and then undergo a single electron transfer from the nitrogen atom of the arylcyclopropylamine to the catalyst (Figure 11A) [58]. More recently, the same compound has also found applications as a robust photoredox catalyst (active at 0.5 mol%) for the difunctionalization of unactivated olefins through the intermolecular addition of α-bromoketones, -esters, or -nitriles, leading to the formation of 4–6 member heterocycles (Figure 11B) [59]. In this case, the catalytic cycle would involve the absorption of a photon by QXPT-NPh, which, in the excited state, would be able to reduce the α-bromoderivative to an alkyl radical species. This latter would then add to the olefin to lead to a radical, which would be oxidized by the oxidized photocatalyst to give a carbocation, which, after entrapment by bromide ions, would undergo a reaction with the nucleophile counterpart [59].

### 3.7. HPLC Detection Systems

**Figure 12:**
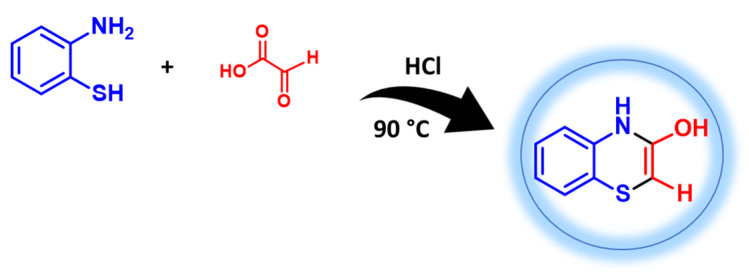
Fluorescent 1,4-benzothiazine derivative through reaction of 2-aminothiophenol with glyoxylic acid in acidic medium [60].

The formation of a fluorescent 1,4-benzothiazine via reaction with 2-aminothiophenol has been exploited for the development of a very sensitive (10 nM detection limit) HPLC method for the quantification of glyoxylic acid in biological fluids (Figure 12). The method was found to be more cost effective and time saving compared to previously reported approaches, requiring a very simple pre-treatment procedure [60].

## 4. Future Directions

From the brief survey presented in this work, it is clear that the 1,4-benzothiazine scaffold is the subject of intense research work today, actually prompted mainly by its pharmacological potential. In particular, several synthetic strategies are still continuously developed and improved, but in the face of numerous efforts in this direction, the possibility to really exploit 1,4-benzothiazines for practical purposes has only rarely been reported. This mainly concerns the opportunities offered by the peculiar spectroscopic, that is, chromophoric and fluorescent properties, of the 1,4-benzothiazine system, for which only scattered examples concerning very structurally different compounds have been described. Although some recent papers have been directed to a detailed investigation of the spectroscopic properties of specific compounds containing 1,4-benzothiazine moieties [61,62], a systematic investigation of the real potential of these compounds is, to the best of our knowledge, still lacking in the literature. Indeed, most of the structure–activity relationship studies available in the literature refer to the biological properties of 1,4-benzothiazines [6,63,64]. In this regard, it is worth noting that the most critical aspects in terms of biological activity seem to be the substitution pattern at the 4- or 1-position, that is, on nitrogen or sulfur atoms [63], whereas in terms of chromophoric and fluorescence properties, the substituents at the 2- and 3-positions affecting the π-electron conjugation properties are apparently the ones playing a major role, at least based on the few available examples reported above.

Another key point that would deserve further attention is the multifunctionality that the 1,4-benzothiazine chromophore may provide. As a remarkable example, the acidichromic behavior of 1,4-benzothiazines conjugated with other chromophoric units has been exploited in pH sensing, smart packaging, and filter permeability modulation/control. If further validated and developed, this aspect would allow the same compound to find applications in various fields also in a sort of “atom economy” perspective. In addition, the possibility to modulate the chromophoric properties of the 1,4-benzothiazine moiety through the introduction of proper substituents, combined with the very easy synthetic accessibility to the 1,4-benzothazine system, may further expand the range of opportunities offered by this class of compounds. On this basis, it would, therefore, be desirable that further research is directed to a detailed and systematic understanding of the whole features and potential of the 1,4-benzothiazine chromophore, in order to rationally conceive new applications for this multifaceted scaffold. This would finally provide the opportunity to take the best from a mutation occurring in nature, which is the one at the basis of phemelanogenesis, whose possible biological benefits are still unknown.
